# Supplementary material for: MetaQTL: a package of new computational methods for the meta-analysis of QTL mapping experiments
Source: BMC Bioinformatics. 2007 Feb 8;8:49. doi: 10.1186/1471-2105-8-49 (PMC1808479; doi:10.1186/1471-2105-8-49)
Supplement: Additional File 7 — MetaQTL Package : jar file and tutorial. This Zip archive contains both the MetaQTL JAR file and the files of the tutorial. [file 1471-2105-8-49-S7.zip › org.inra.metaqtl/doc/org/thalia/bio/class-use/IBioGenome.html]

Uses of Interface org.thalia.bio.IBioGenome


|  |  |  |
| --- | --- | --- |
| **Overview** \| **Package** \| **Class** \| **Use** \| **Tree** \| **Deprecated** \| **Index** \| **Help** | |  |
| PREV   NEXT | **FRAMES**    **NO FRAMES**     **All Classes** |  |


---


## **Uses of Interface org.thalia.bio.IBioGenome**

| Packages that use IBioGenome | |
| --- | --- |
| **org.metaqtl** |  |
| **org.metaqtl.adapter** |  |
| **org.metaqtl.algo** |  |
| **org.metaqtl.factory** |  |
| **org.metaqtl.main** |  |
| **org.metaqtl.util** |  |
| **org.thalia.bio** |  |
| **org.thalia.bio.entity** |  |
| **org.thalia.bio.util** |  |

Uses of IBioGenome in org.metaqtl

| Methods in org.metaqtl with parameters of type IBioGenome | |
| --- | --- |
| `void` | `MapCluster.addAllMap(IBioGenome[] maps)` |
| `void` | `MapCluster.addMap(IBioGenome map, boolean skeleton)`             Add a map to the cluster. |

Uses of IBioGenome in org.metaqtl.adapter

| Methods in org.metaqtl.adapter that return IBioGenome | |
| --- | --- |
| `static IBioGenome` | `ChromosomeAdapter.toIBioGenome(Chromosome[] chromosomes, int gout)`             This routine converts an array of chromosomes into a genetic map with as many chromosomes as the length of the array. |
| `static IBioGenome` | `MetaMapAdapter.toIBioGenome(MetaMap metaMap)`             This methos convert a `MetaMap` object into an object which is consistent with the `IBioGenome` interface. |

| Methods in org.metaqtl.adapter with parameters of type IBioGenome | |
| --- | --- |
| `static Chromosome[]` | `ChromosomeAdapter.toChromosomes(IBioGenome map)`             This methods converts the given map into an array of `Chromosome` which represents the chromosomes of the input mpe. |

Uses of IBioGenome in org.metaqtl.algo

Constructors in org.metaqtl.algo with parameters of type IBioGenome

`ClustAlgorithm(IBioGenome map,
IBioOntology ontology)`

`ClustAlgorithm(IBioGenome map,
java.lang.String name,
IBioOntology ontology)`

`MetaMapAlgorithm(IBioGenome[] maps,
IBioGenome skeleton,
IBioLocus[] dubious,
MetaDico mrkDico)`

`MetaMapAlgorithm(IBioGenome[] maps,
IBioGenome skeleton,
IBioLocus[] dubious,
MetaDico mrkDico)`

`QtlClustAlgorithm(IBioGenome map,
IBioOntology ontology)`

`QtlClustAlgorithm(IBioGenome map,
java.lang.String chrom,
IBioOntology ontology)`

`QtlProjAlgorithm(IBioGenome[] maps,
IBioGenome skeleton,
IBioLocus[] dubious)`
  
          Creates a new instance of `QtlProjAlgorithm` for
the given configuration of projection.

`QtlProjAlgorithm(IBioGenome[] maps,
IBioGenome skeleton,
IBioLocus[] dubious)`
  
          Creates a new instance of `QtlProjAlgorithm` for
the given configuration of projection.

`QtlTreeAlgorithm(IBioGenome map,
IBioOntology ontology)`

Uses of IBioGenome in org.metaqtl.factory

| Methods in org.metaqtl.factory that return IBioGenome | |
| --- | --- |
| `static IBioGenome[]` | `MapDBFactory.read(java.io.Reader reader)`             This method reads a file formated as follows : map property1 property2 property3 ... |

Uses of IBioGenome in org.metaqtl.main

| Methods in org.metaqtl.main that return IBioGenome | |
| --- | --- |
| `static IBioGenome` | `MetaMain.getMap(IBioGenome[] maps, java.lang.String mapName)` |
| `protected static IBioGenome` | `MetaMain.getMap(java.lang.String mapFile)` |
| `protected static IBioGenome[]` | `MetaMain.getMaps(java.lang.String qtlDir)` |

| Methods in org.metaqtl.main with parameters of type IBioGenome | |
| --- | --- |
| `static IBioGenome` | `MetaMain.getMap(IBioGenome[] maps, java.lang.String mapName)` |
| `static void` | `MetaMain.removeLocus(IBioGenome[] maps, java.lang.String mrkRmFile)` |
| `protected static void` | `MetaMain.writeMap(IBioGenome map, java.lang.String outFile)` |
| `protected static void` | `MetaMain.writeMap(IBioGenome genome, java.lang.String format, java.lang.String outFile)` |

Uses of IBioGenome in org.metaqtl.util

| Methods in org.metaqtl.util with parameters of type IBioGenome | |
| --- | --- |
| `static CrossPopulation` | `GeneticMapProperties.getCrossDesign(IBioGenome map)`             This routine returns an object which handle the properties of the offspring which have been used to create the genetic map. |
| `static CrossPopulation` | `GeneticMapProperties.getMappingCross(IBioGenome map)` |
| `static int` | `GeneticMapProperties.getMappingFunction(IBioGenome map)`             This methods returns the integer code of the mapping function associated to the given genetic map. |
| `static int` | `GeneticMapProperties.getMappingUnit(IBioGenome map)` |
| `static boolean` | `GeneticMapProperties.needToBeRescaled(IBioGenome map)`             Returns true if the given map need to be rescaled - due to the mapping extansion for some pedigree designs. |
| `static void` | `GeneticMapProperties.setCrossDesign(IBioGenome map, CrossPopulation crossDesign)` |
| `static void` | `GeneticMapProperties.setMappingContext(IBioGenome map, int mapping_function, int mapping_unit)` |

Uses of IBioGenome in org.thalia.bio

| Methods in org.thalia.bio that return IBioGenome | |
| --- | --- |
| `IBioGenome` | `IBioLGroup.getGenome()` |

| Methods in org.thalia.bio with parameters of type IBioGenome | |
| --- | --- |
| `void` | `IBioLGroup.setGenome(IBioGenome genome)` |

Uses of IBioGenome in org.thalia.bio.entity

| Classes in org.thalia.bio.entity that implement IBioGenome | |
| --- | --- |
| `class` | `GeneticMap`             Class Description Here |
| `class` | `GroupContainer`             Class Description Here |

| Methods in org.thalia.bio.entity that return IBioGenome | |
| --- | --- |
| `IBioGenome` | `LocusContainer.getGenome()` |

| Methods in org.thalia.bio.entity with parameters of type IBioGenome | |
| --- | --- |
| `void` | `LocusContainer.setGenome(IBioGenome genome)` |

Uses of IBioGenome in org.thalia.bio.util

| Methods in org.thalia.bio.util with parameters of type IBioGenome | |
| --- | --- |
| `static void` | `BioUtilities.mergeGenome(IBioGenome g1, IBioGenome g2)`             This method merges two genomes in a single one : `g1` is modified and will contain the result of the merge. |

---


|  |  |  |
| --- | --- | --- |
| **Overview** \| **Package** \| **Class** \| **Use** \| **Tree** \| **Deprecated** \| **Index** \| **Help** | |  |
| PREV   NEXT | **FRAMES**    **NO FRAMES**     **All Classes** |  |


---
